# Piezo-VFETs: Vacuum Field Emission Transistors Controlled by Piezoelectric MEMS Sensors as an Artificial Mechanoreceptor with High Sensitivity and Low Power Consumption

**DOI:** 10.3390/s24206764

**Published:** 2024-10-21

**Authors:** Chang Ge, Yuezhong Chen, Daolong Yu, Zhixia Liu, Ji Xu

**Affiliations:** 1 The Department of Electrical and Computer Engineering, The University of British Columbia, Vancouver, BC V6T 1Z1, Canada; 2 School of Electronic and Information Engineering, Nanjing University of Information Science & Technology, Nanjing 210044, China; 202312180040@nuist.edu.cn (Y.C.); 13731983759@163.com (D.Y.); 202312490430@nuist.edu.cn (Z.L.)

**Keywords:** vacuum emission transistors, piezoelectric vacuum emission transistors, artificial mechanoreceptors, high sensitivity, electrically adjustable measurement range

## Abstract

As one of the most promising electronic devices in the post-Moore era, nanoscale vacuum field emission transistors (VFETs) have garnered significant attention due to their unique electron transport mechanism featuring ballistic transport within vacuum channels. Existing research on these nanoscale vacuum channel devices has primarily focused on structural design for logic circuits. Studies exploring their application potential in other vital fields, such as sensors based on VFET, are more limited. In this study, for the first time, the design of a vacuum field emission transistor (VFET) coupled with a piezoelectric microelectromechanical (MEMS) sensing unit is proposed as the artificial mechanoreceptor for sensing purposes. With a negative threshold voltage similar to an N-channel depletion-mode metal oxide silicon field effect transistor, the proposed VFET has its continuous current tuned by the piezoelectric potential generated by the sensing unit, amplifying the magnitude of signals resulting from electromechanical coupling. Simulations have been conducted to validate the feasibility of such a configuration. As indictable from the simulation results, the proposed piezoelectric VFET exhibits high sensitivity and an electrically adjustable measurement range. Compared to the traditional combination of piezoelectric MEMS sensors and solid-state field effect transistors (FETs), the piezoelectric VFET design has a significantly reduced power consumption thanks to its continuous current that is orders of magnitude smaller. These findings reveal the immense potential of piezoelectric VFET in sensing applications, building up the basis for using VFETs for simple, effective, and low-power pre-amplification of piezoelectric MEMS sensors and broadening the application scope of VFET in general.

## 1. Introduction

Since their emergence, vacuum electronic devices have found vital applications in critical fields such as national defense, aerospace, radio frequency, and terahertz technologies [1,2,3], owing to their superior performance in high-frequency operation [4,5], immunity to radiation [6], and robustness against harsh environments [7,8]. Traditional vacuum electronic devices have issues such as large size, fragility, high manufacturing cost, and low integration capability, significantly limiting their applications in modern communication and integrated circuits. Nonetheless, the theoretical speed of electron transmission in a vacuum channel can reach approximately 3 × 10^10^ cm/s, whereas in semiconductor materials, it is limited to 5 × 10^7^ cm/s [5]. This significant difference endows vacuum electronic devices with an inherent advantage in carrier transport mechanisms and high electron energy, making them irreplaceable in critical applications such as X-ray sources [9] and high-resolution electron microscopy [10].

As micromachining technologies rapidly evolve, it becomes more and more economical to manufacture miniaturized vacuum field emission transistors with nanoscale vacuum gaps [4,5,7,11,12,13,14,15,16]. Moreover, with outstanding carrier transport mechanisms comparable to traditional vacuum electronic devices, these nanoscale vacuum electronic devices have increased on-chip integration capabilities, making them one of the most promising next-generation electronic devices. As revealed by existing studies in the field, nanoscale VFETs have diverse structural designs that can be implemented using a wide range of materials [8,12,13,14,17,18,19]. Some nanoscale VFETs are reported to be capable of operating at a voltage below 30 V [5,7,15,17,18,19]. They also exhibit excellent tolerance against high temperatures and intense radiation [6,7,8,17,20].

Motivated by these experimentally validated outstanding characteristics, existing studies have tried to build functional logic circuits with nanoscale VFETs [8,21,22]. One representative achievement in this respect is the development of complementary vacuum field emission transistors (CVFETs) using VFETs and micro/nanoelectromechanical systems (MEMS/NMES) [23,24]. Han et al. have validated that CVFETs combined with MEMS/NEMS actuators can achieve complementary characteristics for functioning inverters [23]. However, the MEMS/NEMS actuators in these CVFETs are gap-varying capacitive actuators with a suspension height at sub-micron levels, inevitably leading to high manufacturing complexity and a prominent risk of failure related to the electrostatic softening of the mechanical spring and the pull-in effect [25,26,27,28].

Regardless of the issues of CVFETs linked with capacitive gap-varying MEMS structures, the philosophy demonstrated by Han et al. enlightens the idea of developing artificial mechanoreceptors for sensing purposes as bionic electronics by combining VFETs with piezoelectric MEMS sensors. Biological mechanoreceptors for sensing purposes can be equivalented to stress-controlled electronics, with their current output tuned by mechanical input [29]. Existing studies on implementing artificial mechanoreceptors have been trying to couple piezoelectric sensing units with field-effect transistors [30,31,32,33,34,35,36,37,38]. The continuous current of the transistors is adjusted by the voltage output of the piezoelectric sensor, imitating the working mechanisms of the mechanically activated ion channels in biological mechanoreceptors and amplifying the signal resulting from the electromechanical coupling. With as simple as a load resistor, the artificial mechanoreceptor based on piezoelectric sensors and N-channel depletion-mode metal oxide semiconductor field effect transistors (MOSFETs) can achieve high sensitivity that is adjustable by tuning drain-source voltage without extra peripheral circuits [30,31,32,33,39]. However, the continuous current of piezo-FETs using field-effect transistors is at the milliampere level, inevitably leading to significant heat and high power consumption.

Supported by relevant theories, one effective way to address the issue related to artificial mechanoreceptors as bionic sensors is to replace field-effect transistors with VFETs. Through proper structure design, VFETs with a negative threshold voltage can be achieved [11,15,18]. Similar to MOSFETs, by interfacing a piezoelectric sensor between the gate and source electrodes of such a VFET, the continuous current of the VFET can also be tuned for sensing purposes. Typical solid-state field-effect transistors (such as MOSFETs) exhibit higher temperature variations, leading to instability in output voltage and affect sensitivity. Moreover, the static current of solid-state field-effect transistors typically reaches milliampere levels (mA), significantly higher than the microampere level (μA) of VFETs, resulting in higher power consumption. Compared to MOSFETs, VFETs exhibit superior temperature stability, and their static current is one to two orders of magnitude lower [11,15], thereby establishing a solid foundation for using VFETs as an alternative to MOSFET for artificial mechanoreceptors with more stable sensitivity and lower power consumptions.

Motivated by the aforementioned potential of VFETs, this study proposes an artificial mechanoreceptor design based on a vacuum field emission transistor with a negative threshold voltage combined with piezoelectric MEMS sensors (piezo-VFETs). The operating principle and feasibility of the piezo-VFETs were verified, and the crucial factors affecting its characteristics were systematically studied through simulations. Finally, the newly proposed artificial mechanoreceptors based on piezo-VFETs are compared with similar devices using solid-state FETs for key performance metrics, demonstrating the potential of the piezo-VFETs in several application scenarios.

## 2. Structure Design of the VFET and the Piezo-VFETs

### 2.1. Design and Model of the VFET for the Artificial Mechanoreceptor

Piezo-FETs commonly use depletion MOSFETs primarily because they are typically in the linear region at zero gate-source voltage [30,31,32,33,34,35,36,37,38]. This characteristic allows them to effectively handle the small oscillatory signals generated by piezoelectric MEMS sensors, reducing the need for additional bias voltage and thereby minimizing the overall power consumption of the system. Following this classic design approach, we have similarly designed VFETs with a negative threshold voltage.

Finite element electromagnetic simulation software CST Studio Suite (version 2022) was utilized to model the VFETs, with the particle tracking solver employed to calculate the electric field distribution, electron trajectories, and emission current of the VFETs. The simulation environment was set to a complete vacuum to eliminate the influence of factors such as vacuum level on the simulation results, and the boundary conditions were configured as open boundaries. Since the drain-source potential V_ds_ and gate-source V_gs_ could only be applied to ideal conductors, the source, drain, and gate were modeled using perfect electric conductor (PEC) materials. The dielectric material between the gate and source, as well as between the drain and gate, was set to silicon dioxide (SiO_2_) with a dielectric constant of 3.9. A field-induced electron emission model was applied to the emission surface of the source electrode. By adjusting the parameters of CST Studio Suite’s built-in field emission formula (as detailed in Section 5), the field emission characteristics of different materials were defined (in this model, n-type silicon was selected as the emission material), allowing the I-V characteristics of the VFETs to be obtained.

**Figure 1:**
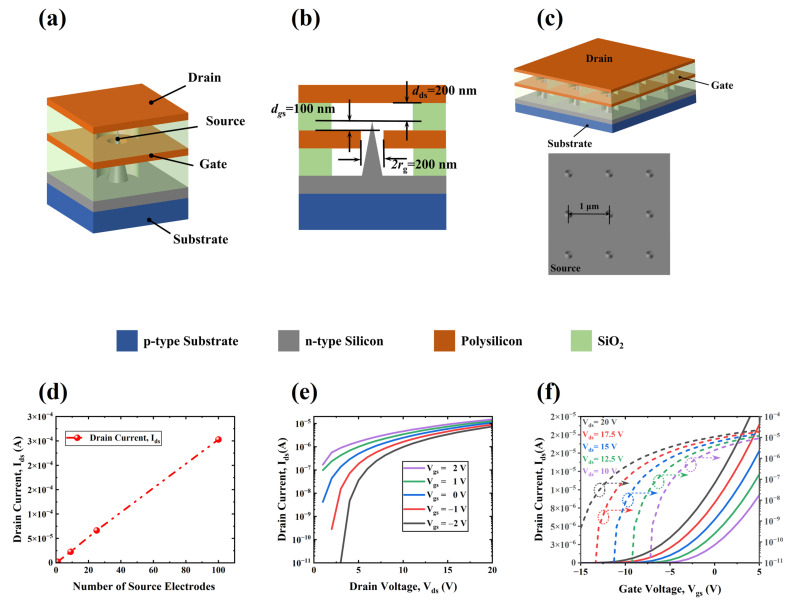
Schematic structure of the proposed VFET: (**a**) overall view; (**b**) cross-sectional view with structural parameters; (**c**) schematic structure of the VFET array; (**d**) the drains current increases linearly with the number of source electrodes. Electrical characteristics of the 3 × 3 VFET array; (**e**) output characteristic curves; and (**f**) transfer characteristic curves.

Figure 1a,b show the schematic structure of the proposed VFET. The sharp source design allows the device to have a lower turn-on electric field [11,12,13]. The gate was placed around the source tip and below the source, facilitating enhanced gate regulation and reduced gate trapping of electrons emitted from the source for a low gate current. More details about the structure design parameters are available in the supporting information.

To enhance the drain current of the VFET, the sources can be typically configured in the form of an array [13,17]. Figure 1c illustrates the schematic structure of a VFET array in which the spacing between the source electrodes is 1 μm. Figure 1d depicts the variation curve of the drain current I_ds_ with the growth of the number of source electrodes at V_ds_ = 20 V and V_gs_ = 5 V. Given that the inter-electrode spacing in the array is sufficiently large, the shielding effect of neighboring fields, which is a consequence of the proximity of the spacings, is effectively reduced, allowing the emitting current of the array VFET to grow linearly with the number of source electrodes, which is consistent with the observation of Han et al. [17]. The following discussions are based on the 3 × 3 VFET array.

Figure 1e,f illustrates the output transfer characteristic curves of the 3 × 3 VFET array. It can be observed that the drain current increases exponentially with a certain external voltage bias, which aligns with the Fowler-Nordheim (F-N) field emission current characteristic [40,41,42,43]. Following the Fowler-Nordheim field emission theory, the field emission current density can be described as follows [44]:

(1) $$J_{FN}=\frac{e^{3}E^{2}}{8\pi h\varphi t(y)}\exp\left(-\frac{8\pi {\left(2m\right)}^{1/2}\varphi^{3/2}}{3heE}v(y)\right)$$

where ***J_FN_*** is the field emission current density (A/m^2^), ***e*** and ***m*** are the charge and mass of the electron, respectively, ***h*** is the Planck’s constant, ***E*** is the source surface electric field (V/m), ***φ*** is the work function of the source, ***y*** is a parametric function of ***E*** and ***φ***, ***t*(*y*)** and ***v***(***y***) are approximated constants. Equation (1) can be simplified as:

(2) $$I_{FN}=\frac{A\alpha \beta^{2}E^{2}}{\varphi }\exp\left(-\frac{B\varphi^{3/2}}{\beta E}\right)$$

where ***I_FN_*** is the emission current, ***A*** = 1.54 × 10^−6^ and ***B*** = 6.83 × 10^7^ are constants [8,18], ***α*** is the emission area (m^2^), and ***β*** is the field enhancement factor, which is related to the shape of the source electrode [45]. Since the electric field ***E*** acting on the surface of the source is due to the combined effect of the drain-source (V_ds_) and gate-source voltage (V_gs_), ***E*** can be expressed as:

(3) $$E=\frac{V_{gs}+\lambda V_{ds}}{d}$$

where ***λ*** is the coefficient that converts V_ds_ into the equivalent stray potential ***λ***V_ds_ associated with the shielding of the gate structure [46], and ***d*** is the vacuum channel distance (m). Furthermore, as ***λ*** is associated with both the external bias of the VFET and its structural parameters, it is frequently challenging to ascertain its value directly. Consequently, it is also possible to determine the value of ***λ*** for the fabricated VFET through the use of an amplification factor ***μ***, which describes the impact of V_gs_ on the emitting current about V_ds_. It can be expressed as follows [11]:

(4) $$\lambda =\frac{1}{\mu }$$

where ***μ*** is:

(5) $$\mu ={\left.-\frac{\partial V_{ds}}{\partial V_{gs}}\right|}_{I_{ds}=constant}$$

The aforementioned ***μ*** can be obtained from the experimentally measured I_ds_-V_ds_-V_gs_ characteristics curve.

For VFET, the electrons are transported in the vacuum channel, eventually reaching the drain as the drain-source current I_ds_. Therefore, I_ds_ can be expressed as:

(6) $$I_{ds}=kI_{FN}$$

Here ***k*** is the transport factor describing the ratio of drain-source current I_ds_ to total emission current ***I_FN_***. On the one hand, it is related to the vacuum level of the device’s operating environment, and on the other hand, under the influence of the gate voltage, the gate traps a part of the electrons emitted from the source, which also affects the ***k*** value. In the simulations in this paper, we set the VFET in a complete vacuum environment to eliminate the influence of environmental factors. In addition, since the gate of the proposed VFET is located below the tip of the source, the electrons captured by the gate can be negligible, and thus the value of ***k*** can be approximated as 1. Eventually, I_ds_ can be expressed by the following equation:

(7) $$I_{\mathrm{ds}}=\frac{A\alpha \beta^{2}{\left(V_{gs}+\frac{V_{ds}}{\mu }\right)}^{2}}{d^{2}\varphi }\exp\left(-\frac{Bd\varphi^{3/2}}{\beta \left(V_{gs}+\frac{V_{ds}}{\mu }\right)}\right)$$

### 2.2. Implementation of the Piezo-VFETs

**Figure 2:**
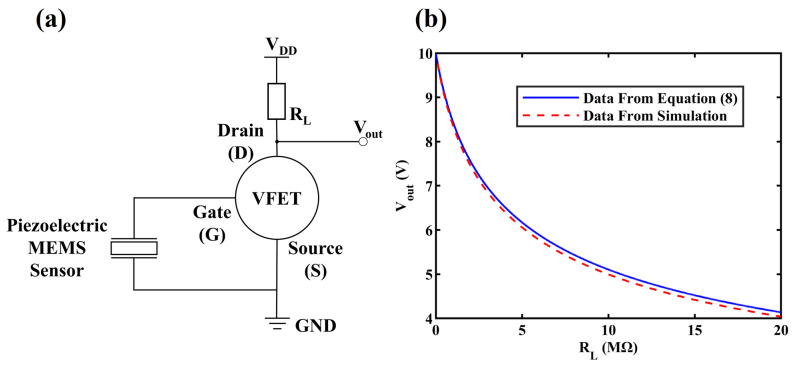
(**a**) Circuit schematic of piezoelectric VFET sensors (piezo-VFETs); and (**b**) the relationship between V_out_ and R_L_ as derived from Equation (8) and as obtained from simulation (V_DD_ = 10 V and V_gs_ = 0 V).

Figure 2a shows the circuit schematic of piezoelectric VFET sensors (piezo-VFETs) consisting of piezoelectric MEMS and a 3 × 3 VFET array. Piezoelectric MEMS sensors are famous for their outstanding capability of measuring dynamic variations of mechanical input [47,48,49,50,51,52,53,54]. The piezoelectric MEMS sensor in Figure 2a is designed as a two-terminal component, with one terminal connected to the source of the proposed VFET and the other terminal connected to the gate of the VFET. A load resistance RL converts the current signal into an output voltage signal, similar to the functionality of a trans-impedance amplifier but with reduced circuit complexity. Unlike directly integrating MEMS/NEMS as part of the VFET structure, piezo-VFETs couple two distinct, discrete components. This approach simplifies the fabrication process and mitigates the risk of irreversible damage to the device due to external factors. Moreover, the fabrication processes for piezoelectric MEMS and VFETs can be carried out separately, ensuring greater control over the manufacturing process and higher yield rates, enhancing both feasibility and efficiency.

For the piezo-VFETs in Figure 2a, an external mechanical input would induce a variation in V_gs_ (ΔV_gs_), which further leads to variations in I_ds_ (ΔI_ds_) and V_out_ (ΔV_out_) to implement the sensing process. Based on Equation (7), the input-output relationship can be expressed as:

(8) $$\begin{array}{l} V_{out}=V_{ds}=V_{DD}-V_{R}=V_{DD}-I_{ds}R_{L}\\ =V_{DD}-\left\{\frac{A\alpha \beta^{2}{\left(V_{gs}+\frac{V_{ds}}{\mu }\right)}^{2}}{d^{2}\varphi }\exp\left(-\frac{Bd\varphi^{3/2}}{\beta \left(V_{gs}+\frac{V_{ds}}{\mu }\right)}\right)\right\}\times R_{L} \end{array}$$

Based on the I_ds_-V_ds_-V_gs_ characteristic curves of the 3 × 3 VFET, as depicted in Figure 1e,f, the calculated ***μ*** value is approximately 1.6. By taking the natural logarithm of both sides of Equation (2), a relationship between ***ln* (I_ds_/E)** and **−1/E^2^** can be obtained, forming the F-N plot [55]. From the slope and intercept of the fitted F-N plot, the values of ***α*** and ***β*** are determined to be 9.81 × 10^−19^ m^2^ and 44.72, respectively.

To verify the accuracy of the derived relationship between the load resistance R_L_ and output voltage V_out_ in the absence of input (i.e., V_gs_ = 0 V) as given in Equation (8), we extracted the VFET model parameters, parasitic elements such as inter-electrode capacitance, and I-V characteristics from the CST Studio Suite simulation. These were then imported into LTspice (version XVII) to build a circuit model and simulate the relationship between load resistances and output voltages. Figure 2b illustrates the relationship between V_out_ and R_L_ as derived from Equation (8) and as obtained from simulation under the V_DD_ = 10 V and V_gs_ = 0 V. The theoretical and simulated curves are mostly consistent, with minor discrepancies attributable to internal errors arising during the extraction of parameters such as inter-electrode capacitance between the multiple simulation tools, as well as the potential inaccuracies in the calculation of ***μ***. This consistency indicates that integrating VFETs with piezoelectric MEMS sensors is theoretically feasible.

## 3. Characteristics of the Piezo-VFETs as Artificial Mechanoreceptors for Sensing

With the feasibility of the structure configuration validated by the simulation, this section further discusses the characteristics of the piezo-VFETs proposed in Section 2 as artificial mechanoreceptors for sensing purposes.

### 3.1. Sensitivity Adjustment Through Parameter Modifications

As per Equation (8), the output voltage of the piezo-VFETs is a function of V_DD_ and R_L_, demonstrating the potential controllability over the output voltage of the artificial mechanoreceptor, which could be considered a significant advantage of integrating piezoelectric MEMS sensors with VFETs. In detail, for a given ΔV_gs_, the amplification ratio of the piezoelectric potential, i.e., ΔV_out_/ΔV_gs,_ can be optimized by selecting appropriate load resistors R_L_ and external DC power supplies V_DD_. This amplification ratio can also be referred to as the sensitivity of the piezo-VFETs.

**Figure 3:**
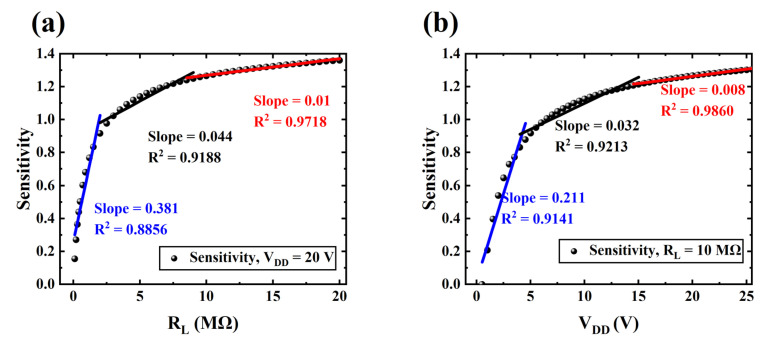
(**a**) Sensitivity as a function of load resistance (R_L_) with a DC power supply voltage (V_DD_) of 20 V; and (**b**) sensitivity as a function of V_DD_ with R_L_ set to 10 MΩ.

Figure 3 illustrates the sensitivity curves of piezo-VFETs with various V_DD_ and R_L_. In general, the sensitivity curve of a sensor requires a high degree of linearity. Compared to capacitive MEMS sensors, one of the primary advantages of piezoelectric MEMS sensors is their ability to maintain high linearity while also achieving high sensitivity, which has contributed to their increasing popularity in recent years [47,48,49,54,56]. Both Figure 3a,b can be divided into three distinct segments. The linear fitting of these segments shows that the sensitivity adjustment of the proposed piezo-VFETs by V_DD_ and R_L_ is highly linear.

Figure 3a shows the ratio versus R_L_ with a constant input signal ΔV_gs_ and V_DD_. The entire curve can be divided into three regions. Initially, before 2.5 MΩ, the curve exhibits a rapid rising phase, where sensitivity to the R_L_ changes significantly. The second region, from 2.5 MΩ to 10 MΩ, represents the sub-saturation zone, where the sensitivity to R_L_ changes gradually diminishes and the trend becomes less pronounced. Finally, beyond 10 MΩ, the sensitivity to R_L_ changes becomes increasingly negligible and stabilizes.

Figure 3b illustrates the sensitivity curve as a function of V_DD_ with a fixed V_gs_ and R_L_ of 10 MΩ. Similar to Figure 3a, the sensitivity curve exhibits three distinct phases as V_DD_ varies. Firstly, before 2.5 V, the sensitivity curve exhibits a rapid increase, with a significant rise in sensitivity to V_DD_. In the second region, ranging from 2.5 V to 15 V, the curve enters a sub-saturation zone. Within this range, the sensitivity response to changes in V_DD_ gradually diminishes, and the trend becomes less pronounced. Finally, when V_DD_ exceeds 15 V, the sensitivity to its variations becomes exceedingly minor and stabilizes.

As shown in Figure 3, the variations in V_DD_ and R_L_ have different degrees of impact on the sensitivity, which is reflected by the slope differences between each segment in Figure 3a,b. Across all three segments, the slope of the sensitivity curve concerning R_L_ is consistently greater than that of V_DD_. Specifically, in the first segment, the slope for R_L_ exceeds that for V_DD_ by approximately 80.56%; in the second segment, it is around 37.5% higher; and in the third segment, it is about 25% greater. This difference suggests that, in practical applications, selecting and optimizing R_L_ is more critical than adjusting V_DD_ to achieve optimal performance and stability of the piezo-VFETs.

Resistive components unavoidably generate Johnson-Nyquist noise [57], which is directly proportional to the resistance value. Consequently, the magnitude of system noise is directly proportional to the system resistance. Additionally, the power consumption of the system, influenced by V_DD_, must also be taken into consideration. Therefore, in practical applications, it is essential to balance sensitivity, noise, and power consumption by selecting appropriate values of V_DD_ and R_L_ to achieve optimal system performance.

### 3.2. Measurement Range Adjustable from the Power Supply and Load Resistance

Generally, the linear measurement range is a critical performance parameter for MEMS sensors. The artificial mechanoreceptors based on the piezo-VFETs proposed here work around the static point of V_gs_ = 0. Its measurement range of mechanical signals is mainly determined by the linearity of the I_ds_-V_gs_ curve of the VFET design in Figure 1. In the transfer characteristic curves of the proposed VFET, as shown in Figure 1f, the variation of I_ds_ with V_gs_ can be broadly divided into two segments with different slopes, where the inflection point between these two segments corresponds not only to the VFET’s threshold voltage but also to the measurement range of the piezo-VFETs. According to Equation (8), a change in V_gs_ (ΔV_gs_) results in a corresponding change in I_ds_ (ΔI_ds_). However, since the mechanical signals ΔV_gs_ generated by most piezoelectric MEMS sensors are smaller than V_ds_ [39,47,48,49,51,53,54], the change in I_ds_ due to V_gs_ is negligible. Consequently, the measurement range of piezo-VFETs can be approximately determined based on Figure 1f. In this figure, different V_ds_ values lead to different inflection points, altering the measurement range of piezo-VFETs.

**Figure 4:**
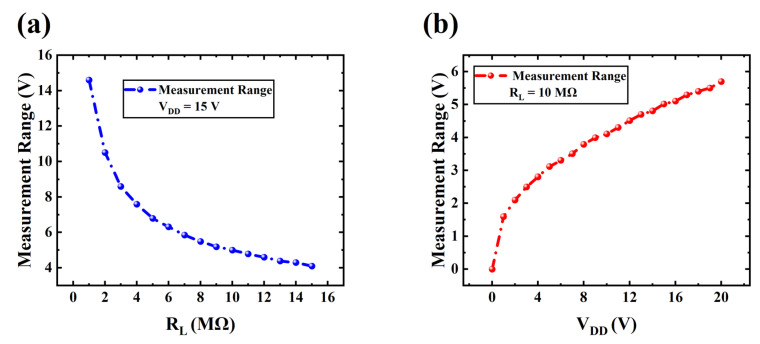
(**a**) Measurement range as functions of R_L_ with V_DD_ set at 15 V; and (**b**) measurement range as functions of V_DD_ with and R_L_ fixed at 10 MΩ.

As inferable from the discussion above, the linear measurement range of an artificial mechanoreceptor proposed in this paper is also affected by R_L_ and V_DD_, both of which control the detail V_ds_ of the VFET. Figure 4a shows the relationship between the measurement range and R_L_ under a V_DD_ of 15 V, illustrating that the measurement range of piezo-VFETs decreases as R_L_ increases. Additionally, as discussed in Section 3.1, while an increase in R_L_ leads to a rise in sensitivity, it also inevitably causes an increase in the overall noise of the piezo-VFETs. This implies that in practical applications, a balance must be struck between measurement range, noise, and sensitivity to select the appropriate load resistor, thereby maximizing overall performance.

Figure 4b illustrates the measurement range curve as a function of V_DD_ with R_L_ of 10 MΩ. Unlike the influence of R_L_ on measurement range, the measurement range increases with the rise of V_DD_. Moreover, as noted in the discussion of the relationship between V_DD_ and sensitivity in Section 3.1, increasing V_DD_ can enhance sensitivity within certain limits, but this also inevitably increases the overall power consumption of the system. Therefore, selecting the appropriate R_L_ and V_DD_ based on different application requirements is necessary to achieve optimal performance.

### 3.3. Impact of VFET Array on the Characteristics of the Piezo-VFETs

**Figure 5:**
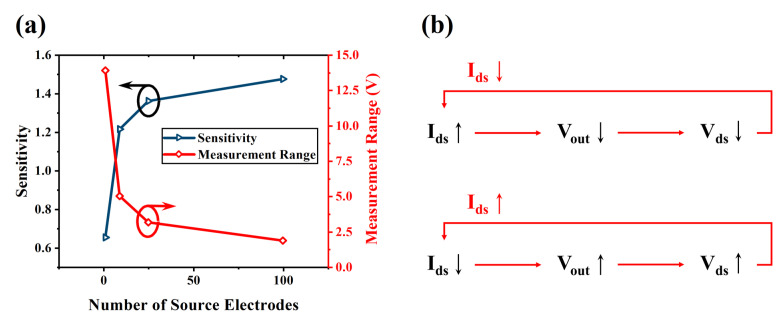
(**a**) Curves depicting sensitivity and measurement range vs. number of source electrodes (V_DD_ = 15 V, R_L_ = 10 MΩ); and (**b**) negative feedback loop (An arrow up refers to rise in current or voltage, while an arrow down refers to a fall in current or voltage.).

As mentioned earlier, forming VFET into arrays can effectively increase the overall operational current. According to Equation (8), for given V_DD_ and R_L_, (given V_DD_ = 15 V and R_L_ = 10 MΩ), changes in I_ds_ will also affect the output voltage V_out_. Figure 5a illustrates the relationship between the number of source electrodes and the sensitivity and measurement range of piezo-VFETs. As shown in Figure 5a, with the increase in the number of source electrodes, the overall output sensitivity of piezo-VFETs also increases, while the measurement range decreases correspondingly. As the number of source electrodes increases, the total current I_ds_ rise under the same external bias conditions, leading to more pronounced changes in output voltage, thereby enhancing sensitivity. This phenomenon occurs because the increase in source electrodes results in each electrode contributing more current. The cumulative effect makes the output signal more responsive to input changes.

Simultaneously, according to Equation (8), when I_ds_ increases, V_out_ decreases. This voltage drop inhibits further increases in I_ds_, thereby forming a negative feedback loop, as shown in Figure 5b. In this process, the system automatically adjusts to reach a new equilibrium point for V_out_ (V_ds_). A change in this equilibrium point leads to a reduction in the measurement range. This is due to the rapid rise in current I_ds_ with the increase in the number of source electrodes causing V_out_ to reach equilibrium more quickly, thus limiting the variable range of V_gs_. This negative feedback mechanism ensures system stability. It also implies a trade-off between sensitivity and measurement range in practical applications. To achieve optimal performance, the appropriate VFET array configuration must be selected based on specific application requirements. In applications requiring high sensitivity, more electrodes may be needed. In applications requiring a larger measurement range, the number of source electrodes should be carefully controlled.

### 3.4. Bandwidth of the Piezo-VFETs

**Figure 6:**
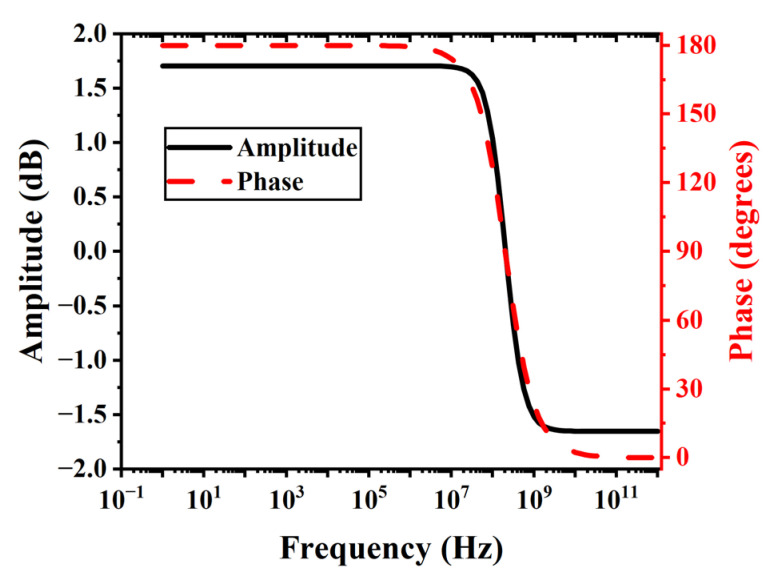
Frequency response characteristic curve (Bode plot) of the piezo-VFETs.

Due to the ballistic transport of electrons within its internal vacuum channel, VFET exhibits excellent frequency response characteristics [4,5,8,15,21]. When integrated with piezoelectric MEMS sensors to amplify the electrical signal output from piezoelectric MEMS sensors, the frequency response characteristic curve of the VFET circuit determines the bandwidth of the entire system. Ideally, the flat band of the VFET circuit should encompass the mechanical resonance frequency of the piezoelectric MEMS sensors. This ensures that the operational bandwidth of the entire system is not adversely affected by the integration. Figure 6 depicts the simulated frequency response characteristic curve (Bode plot) of the piezo-VFETs at V_DD_ = 15 V and R_L_ = 10 MΩ. It can be seen that the flat band of the VFET circuit covers frequencies below 10 MHz. Table 1 lists the frequency bandwidth of representative piezoelectric MEMS sensors. As inferable from Table 1, the VFET circuit has a flat band encompassing the mechanical resonance frequency of most piezoelectric MEMS sensors, demonstrating that the piezo-VFETs proposed in this paper can be effectively used to couple with piezoelectric MEMS sensors to form artificial mechanoreceptors for sensing purposes [47,48,49,50,51,56,58,59,60].

### 3.5. Noise of the Piezo-VFETs as an Artificial Mechanoreceptor

For the piezo-VFETs proposed in this paper, the primary noise source is the piezoelectric sensing unit. The noise of the piezoelectric sensing unit can be written as follows [62]:

(9) $$\left\{\begin{array}{c} v_{n}=\sqrt{4k_{B}T\left(\frac{\omega_{0}Q_{T}^{2}}{C^{2}mQ}+\frac{\eta }{\omega C}\right)}\\ V_{n}=v_{n}\sqrt{\Delta f} \end{array}\right.$$

Here, ***v*_n_** represents the total voltage noise density of the piezoelectric sensing unit (V/√Hz), ***k*_B_** is Boltzmann’s constant (1.38 × 10^−23^ J/K), **T** is the absolute temperature (K), **ω_0_** is the angular mechanical resonant frequency, ***Q*_T_** is the piezoelectric sensing unit’s charge sensitivity, ***C*** is the total capacitance of the piezoelectric sensing unit, ***m*** is the mass (kg), ***Q*** is the quality factor, ***η*** is the dissipation constant of the piezoelectric material, ***ω*** is the angular frequency, ***V*_n_** is the total voltage noise (V), and **Δ*f*** is the bandwidth in hertz (Hz) over which the noise is measured.

Based on Equation (9), the noise reflected in the V_out_ of the piezo-VFETs is:

(10) $$V_{out}=V_{DD}-\left\{\frac{A\alpha \beta^{2}{\left(V_{n}+V_{gs}+\frac{V_{ds}}{\mu }\right)}^{2}}{d^{2}\varphi }\exp\left(-\frac{Bd\varphi^{3/2}}{\beta \left(V_{n}+V_{gs}+\frac{V_{ds}}{\mu }\right)}\right)\right\}\times R_{L}$$

The noise generated by the piezoelectric sensing unit is coupled to the VFETs through the input voltage, subsequently affecting the overall output noise level of the piezo-VFETs. This process indicates that the noise performance of the piezoelectric sensing unit directly determines the overall noise behavior of the piezo-VFETs, particularly in high-sensitivity applications where noise amplification effects are more pronounced. To mitigate the noise issue, structural optimization of the piezoelectric sensing unit is crucial. Adjustments in the design and material selection of the piezoelectric unit can reduce intrinsic noise. For instance, improving the uniformity of the piezoelectric material can help minimize noise coupling into the VFETs. Additionally, electromagnetic shielding and incorporating appropriate filtering circuits can further reduce noise in the entire system. In the final integration of the VFETs and piezoelectric sensing unit, the use of appropriate vacuum packaging to place the system in an optimal working environment can further reduce the impact of external environmental noise on the piezo-VFETs, thereby enhancing the overall stability and measurement accuracy of the system.

## 4. Application Potential of Piezo-VFETs as Artificial Mechanoreceptors for Sensing Purposes

The characteristics of the newly proposed piezo-VFETs revealed in Section 3 indicate its promising potential. For further clarification, this section uses polymeric piezoelectric accelerometers in existing studies as examples to show the significance of the piezo-VFETs proposed in this paper.

### 4.1. Increment of Sensitivity, Bandwidth, and Degree of Miniaturization

**Figure 7:**
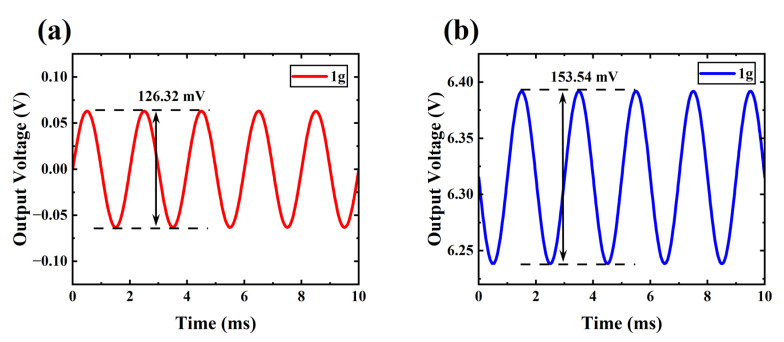
The output characteristic of PVDF-based piezoelectric MEMS accelerometers (**a**) piezo-VFETs; and (**b**) under a 1g acceleration (V_DD_ = 15 V, R_L_ = 10 MΩ).

In 2023, Ge et al. proposed a PVDF-based piezoelectric MEMS accelerometer with a voltage sensitivity of 126.32 mV/g (1g = 9.8 m/s^2^) [47]. If this polymer piezoelectric accelerometer is interfaced with a VFET array in Figure 1 to form an artificial mechanoreceptor based on piezo-VFETs (V_DD_ = 15 V and R_L_ = 10 MΩ), as shown in Figure 7, the voltage output is increased by 1.216 times.

Traditional piezoelectric MEMS sensors have their sensitivity positively related to the planar dimensions of the piezoelectric layers sandwiched by electrodes [47,49,50,58,60]. For polymeric piezoelectric accelerometers such as the ones of Ge et al., due to the inferior piezoelectric properties of polymers, their accelerometers have very limited degrees of miniaturization and bandwidth. As indicated by the sensitivity increment in Figure 7, using such polymeric piezoelectric MEMS sensors in the newly proposed piezo-VFETs allows MEMS devices to achieve further miniaturization and, correspondingly, increased bandwidth while maintaining consistent sensitivity. Moreover, compared with the classic piezoelectric MEMS readout circuit based on charge amplifiers using operational amplifiers, the complexity of the readout circuit of the piezo-VFETs proposed in this paper is also reduced [47,48].

### 4.2. Reduced Power Consumption than Piezo-FETs

VFETs inherently have a lower continuous current than most solid-state field effect transistors (FETs), which translates to a distinct advantage in power consumption, making the integration of piezoelectric MEMS sensors with VFETs more advantageous than with other solid-state FETs.

For the VFET array combined with the polymer piezoelectric MEMS accelerometers of Ge et al., simulations estimate its static power consumption to be 83.7 μW at V_DD_ = 15 V and R_L_ = 10 MΩ. Table 2 summarizes the continuous current, corresponding power consumption, fabrication complexity, and cost of piezo-FETs as sensors in existing studies [33,34,35,36,37,38,63,64,65,66], with a comparison to our piezo-VFETs, using Ge et al.’s polymer piezoelectric MEMS accelerometers as an example. We evaluate the fabrication complexity based on its compatibility with modern integrated circuits and Complementary Metal-Oxide-Semiconductor (CMOS) processes, as well as the integration difficulty with piezoelectric sensing units. Additionally, the fabrication cost is assessed through the complexity of the fabrication process, the difficulty of large-scale production, and material costs.

As shown in Table 2, although some solid-state FETs fabricated with advanced materials such as graphene exhibit lower power consumption than VFETs, when silicon is used as the semiconductor material, the continuous current of such solid-state FETs is generally in the milliampere range, and their power consumption is in the microwatt range. In contrast, the continuous current of our silicon based VFET is reduced by an order of magnitude, highlighting its advantage in power efficiency. Additionally, solid-state FETs suffer from poor thermal stability, with their continuous current significantly affected by temperature environments. Although directly integrating the piezoelectric sensing unit as part of solid-state FETs, such as using it as the gate of FETs, allows for higher miniaturization, this approach faces more complex fabrication processes and is challenging to achieve large-scale production. Moreover, stability and reliability remain concerns. Separating the fabrication of the piezoelectric sensing unit and VFETs can avoid compatibility issues in the fabrication process, thus improving the stability of piezo-VFETs as artificial mechanoreceptors and reducing manufacturing costs. Overall, this highlights the clear advantages of VFETs in terms of low power consumption. Thermal stability and fabrication cost make them an ideal choice for integration with piezoelectric MEMS sensors.

## 5. Method

All the data presented in this study were obtained through simulations, using a combined approach with the finite element electromagnetic simulation software CST Studio Suite (version 2022) and the circuit simulation software LTspice (version XVII). First, the particle tracking solver in CST Studio was utilized to design the VFET structure and calculate its electrical characteristics. The simulation environment was set to a complete vacuum to eliminate the influence of the external environment, thereby achieving more accurate results. The Fowler-Nordheim (F-N) field emission model was employed in the CST Studio Suite, and Equation (1) was simplified as follows:

(11) $$J_{FN}=a_{FN}E^{2}\exp\left(-b_{FN}/E\right)$$

Based on the field emission parameters of silicon-based material obtained from our previous experiments, the values of ***a*_FN_** = 6.0 × 10^−9^ (unit: A/V^2^) and ***b*_FN_** = 2.56 × 10^7^ (unit: V/m) were used in the simulation. Subsequently, the VFET structure parameters, I-V characteristics, and parasitic parameters such as inter-electrode capacitance was extracted from CST Studio Suite and imported into LTspice to construct the VFET equivalent circuit model. This model facilitated further analysis of the piezo-VFETs’ characteristics.

## 6. Conclusions

In this paper, a novel vacuum field emission transistor design was proposed. It was customized to integrate piezoelectric MEMS sensors to form piezo-VFETs for artificial mechanoreceptors as bionic electronics. Theoretical studies and simulations were conducted to validate the feasibility of such a configuration and to demonstrate its characteristics. The corresponding results reveal that the sensitivity and measurement range of the newly proposed piezo-VFETs can be optimized by adjusting the load resistance and DC power supply. Additionally, the simulation results indicated that the sensitivity and measurement range of piezo-VFETs can be effectively tuned by varying the number of source electrodes in the VFET array. Moreover, the superior high-frequency performance of the VFET was found to align with the flat-band resonance frequency of the MEMS devices, ensuring that the integration of the two components is not constrained by bandwidth limitations. In a case study based on polymeric piezoelectric MEMS accelerometers interfaced with the classic charge amplifier circuits, it is demonstrated that the sensitivity of piezo-VFETs was increased by approximately 1.2 times compared to the original piezoelectric MEMS sensor with a significantly simplified readout circuit. In addition, the simulation in this paper also demonstrates that, compared to piezo-FETs using field-effect transistors, the piezo-VFETs illustrate a significant advantage in power consumption. This paper underscores the significant potential of piezoelectric VFETs in high-performance sensor applications, laying a solid foundation for the broader integration of piezoelectric VFETs in the future.

## Figures and Tables

**Table 1:** The frequency bandwidth of representative piezoelectric MEMS sensors.

Research	Piezoelectric Material	Sensor Types	Bandwidth	Resonance Frequency
Ali et al. [60]	ZnO	Acoustic sensor	15 kHz	78 kHz
Prasad et al. [61]	ZnO	Acoustic sensor	8 kHz	42.875 kHz
Minh et al. [59]	(K, Na) NbO_3_	Energy harvester	150 Hz	3.094 kHz
Ge et al. [47]	PVDF	Accelerometer	58.6 Hz	128.95 Hz
Gong et al. [49]	PZT	Accelerometer	200 Hz	857.4 Hz
Yang et al. [58]	AlN	Hydrophone	2 kHz	479 kHz

**Table 2:** The continuous current, corresponding power consumption, fabrication complexity, and cost of piezo-FETs as sensors in existing studies with a comparison to our piezo-VFETs.

Researchers	Sensor Types	Semiconductor Material	Continuous Current	Sensitivity	Operating Voltage	Power Consumption	Fabrication Complexity	Cost
Dahiya et al. [63]	Tactile	Silicon	3~4 mA	50 mV/N (Load: 100 kΩ)	V_SS_ = −5 V	15~20 mW	Middle	Low
Maita et al. [64]	Tactile	Silicon	~40 µA	430 mV/N (Load: 270 kΩ)	V_DD_ = 20 V, V_G_ = 9 V	~0.8 mW	Middle	Low
Viola et al. [65]	Tactile	Polyethylene naphthalene (PEN)	−3~−4 µA	0.15 nA/kPa	V_DD_ = V_G_ = −2 V	6~8 µW	Easy	Low
Wang et al. [33]	Tactile	InSe	~1 mA	0.1 mA/N	V_ds_ = 1 V	~1 mW	Difficult	High
Wang et al. [32]	Tactile	Pentacene	1~2 µA	21 nA/N	V_ds_ = −40 V	40~80 µW	Middle	High
Yogeswaran et al. [30]	Tactile	Graphene	8~10 µA	2.7 × 10^−4^ kPa^−1^ (ΔI/I_0_)	V_ds_ = 50 mV	0.4 µW	Difficult	High
Hsu et al. [66]	Strain gauge	Pentacene	~100 nA	0.182 µA/strain	V_ds_ = 35 V	3.5 µW	Easy	Low
Zhu et al. [35]	Strain gauge	Silicon	~1 mA	1340 ppm	V_ds_ = 3 V, V_G_ = 3.15 V	~3 mW	Middle	Middle
Ueda et al. [37]	Inertial	ZnO	~10 µA	N/A	V_ds_ = 0.1 V	~1 µW	Easy	Low
Ai et al. [39]	Inertial	Silicon	~100 mA	2.05 V/g	V_DD_ = 5 V	500 mW	Easy	Low
This work	Inertial	Silicon	~5.58 µA	1.216 V/g	V_DD_ = 15 V	83.7 µW	Easy	Middle

## Data Availability

All data that support the findings of this study are included within the article (and any Supplementary Materials).

## Supplementary Materials

The following supporting information can be downloaded at: https://www.mdpi.com/article/10.3390/s24206764/s1, Figure S1: (a) Structure parameters of proposed VFET, highlighting the gate-source distance (*d_gs_*), drain-source distance (*d_ds_*), and gate radius (*r_g_*). (b) Transfer characteristic curves with various *d_gs_* (V_ds_ = 20 V, and *r_g_* = 100 nm). Electric field distribution at source tip for different *d_gs_* values: (c)*d_gs_* = 100 nm, (d) *d_gs_* = 25 nm, (e) *d_gs_* = −25 nm, and (f) *d_gs_* = −100 nm.; Figure S2: (a) The structure parameters of the proposed VFET highlight the gate-source distance (*d_gs_* = 100 nm), drain-source distance (*d_ds_* = 200 nm), and gate radius. (b) Transfer characteristic curves with various combinations of gate radii (V_ds_ = 20 V, and *d_gs_* = 100 nm); Figure S3: Schematics (not to scale) showing the Outlines of the fabrication process of the proposed VFET.
