# Supplementary material for: Piezo-VFETs: Vacuum Field Emission Transistors Controlled by Piezoelectric MEMS Sensors as an Artificial Mechanoreceptor with High Sensitivity and Low Power Consumption
Source: Sensors (Basel). 2024 Oct 21;24(20):6764. doi: 10.3390/s24206764 (PMC11511378; doi:10.3390/s24206764)
Supplement: Supplementary file 1 [file sensors-24-06764-s001.zip › sensors-3238096-supplementary.pdf]

Supporting Information

1. Influence of Structural Parameters on VFET Threshold Voltage

To further investigate the influence of gate position and structural parameters on the performance of VFET, the transfer characteristic curves as a function of the gate-source distance (d_{gs}) are depicted in **Figure S1(b)**, with a gate radius (r_g) of 100 nm. Here, a positive d_{gs} indicates that the gate is below the source tip, while a negative d_{gs} implies that the gate is above the source tip. It can be observed from **Figure S1(b)** that when the gate is positioned below the source tip, the threshold voltage shifts toward more negative values. This results in a steeper current slope at V_{gs}=0 V, leading to higher sensitivity and a wider measurement range when combined with piezoelectric MEMS sensors. **Figure S1(c)** to **Figure S1(f)** illustrate the electric field distributions for different d_{gs} values (V_{ds}=20 V, V_{gs}=5 V). The figures clearly show that as the gate is positioned lower relative to the source tip, the electric field at the source tip becomes more concentrated, resulting in a higher field intensity. This phenomenon can be attributed to two main factors: firstly, the bending of electric field lines when the gate is below the source, which causes the field to concentrate at the source tip; and secondly, the reduced shielding effect of the gate structure on the electric field exerted by the drain on source, allowing the field at the source tip to be more strongly influenced by the drain.

We explored the effects of varying gate radii to investigate further the relationship between gate radius and threshold voltage under d_{gs} =100 nm. Here, r_g*_bottom* denotes the bottom gate radius, and r_g*_top* represents the top gate radius. **Figure S2(b)** illustrates the transfer characteristics for various combinations of gate radii. It can be observed that the gate radius has minimal impact on the threshold voltage, showing relatively stable behavior. Consequently, considering the practical challenges of fabrication, a gate radius of 100 nm was chosen for both r_g*_bottom* and r_g*_top* for subsequent analysis.

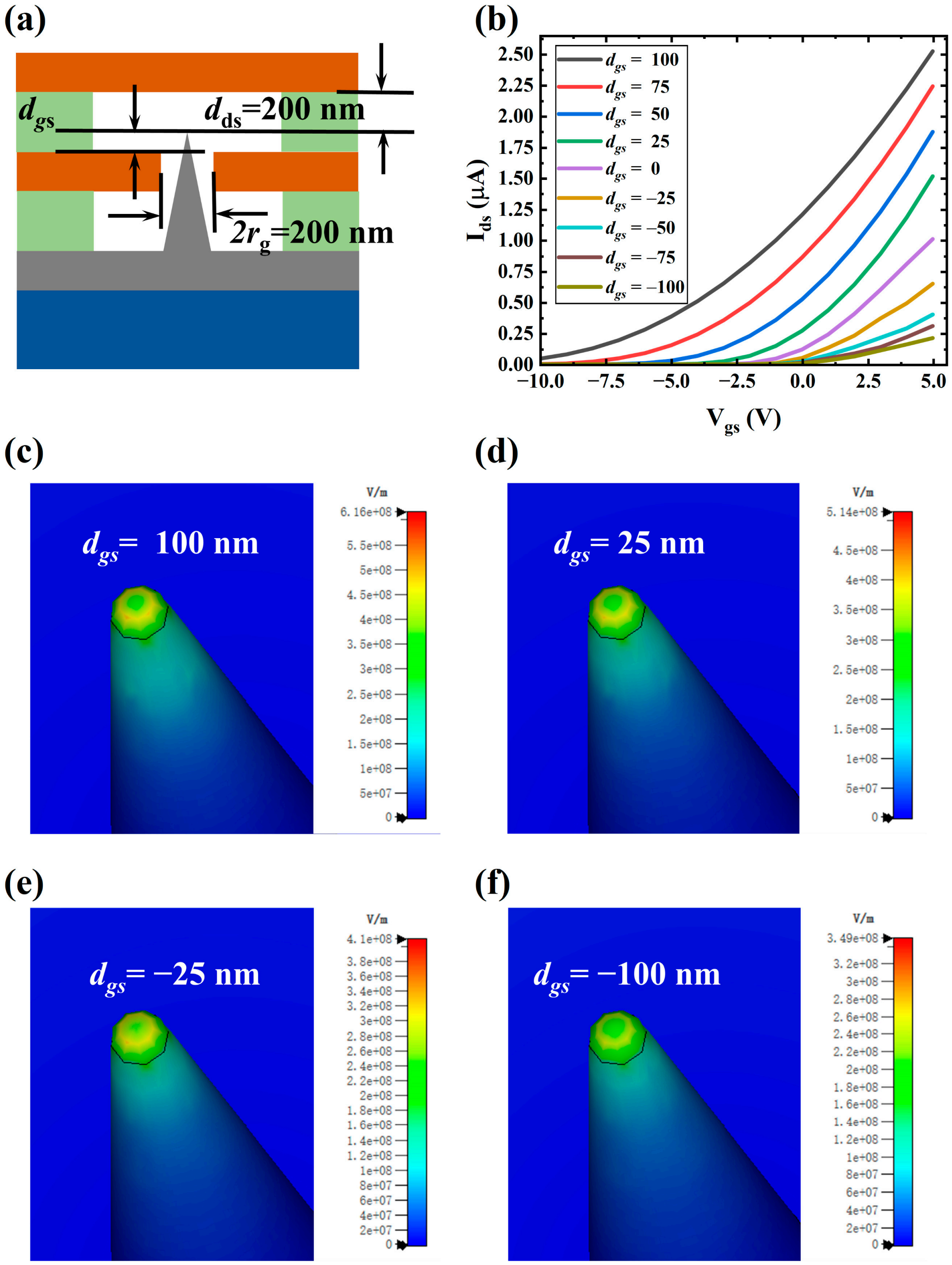

Figure S1. (a) Structure parameters of proposed VFET, highlighting the gate-source distance (d_{gs}), drain-source distance (d_{ds}), and gate radius (r_g). (b) Transfer characteristic curves with various d_{gs} (V_{ds}=20 V, and r_g=100 nm). Electric field distribution at source tip for different d_{gs} values: (c)d_{gs}=100 nm, (d) d_{gs}=25 nm, (e) d_{gs}= −25 nm, and (f) d_{gs}= −100 nm.

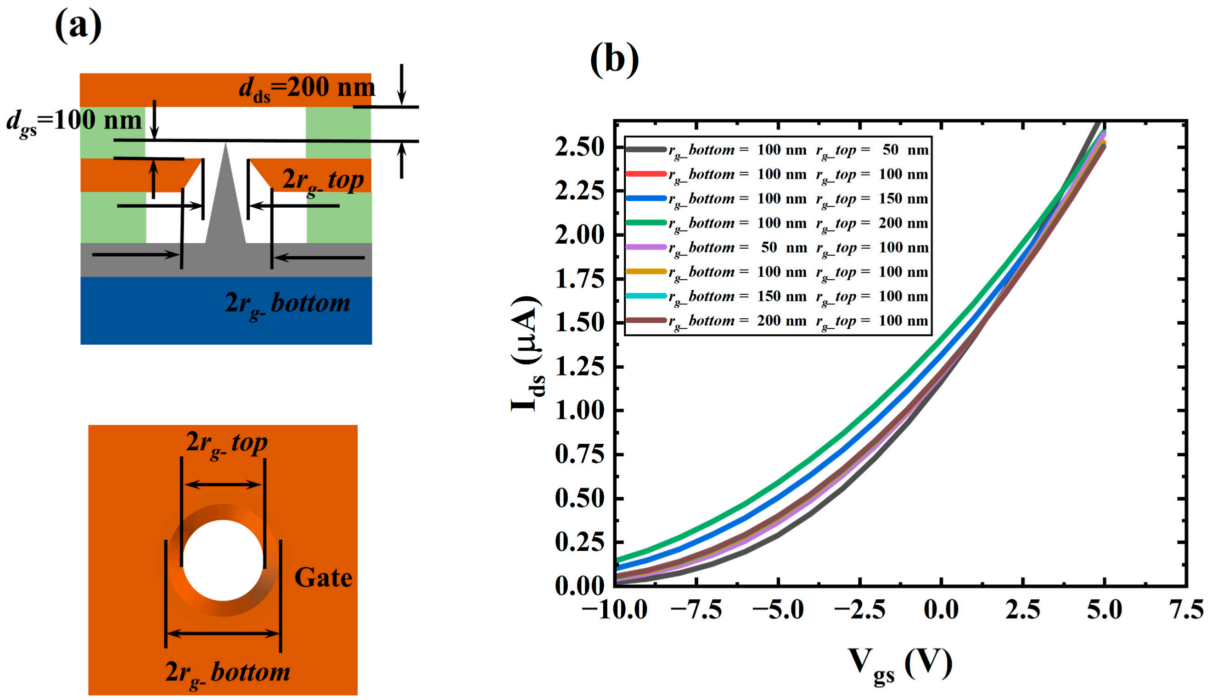

Figure S2. (a) The structure parameters of the proposed VFET highlight the gate-source distance (d_{gs}=100 nm), drain-source distance (d_{ds}=200 nm), and gate radius. (b) Transfer characteristic curves with various combinations of gate radii (V_{ds}=20 V, and d_{gs}=100 nm).

2. Potential Process Flow for Vacuum Field Emission Transistor Fabrication

Figure S3 illustrates a potential fabrication process for the proposed VFET. A naturally grown thin oxide layer forms on the n-type silicon substrate in air. The sample is initially cleaned, followed by the formation of a photoresist mask on the sample surface using photolithography. This mask is employed for the preliminary etching of silicon pillars in the second step, thereby defining the approximate morphology of the source electrode. In the third and fourth steps, anisotropic etching techniques further process the silicon pillars to create sharp features at the source electrode tip and remove residual oxide layers. The gate structure is then fabricated through oxide and gate material deposition and precise etching in the fifth and sixth steps. The seventh and eighth steps focus on the preparation of the drain electrode. Initially, a silicon dioxide layer is deposited and etched to form a shape similar to a vacuum cavity[1,2]. Finally, a tilt deposition process is used to accurately fabricate the drain electrode.

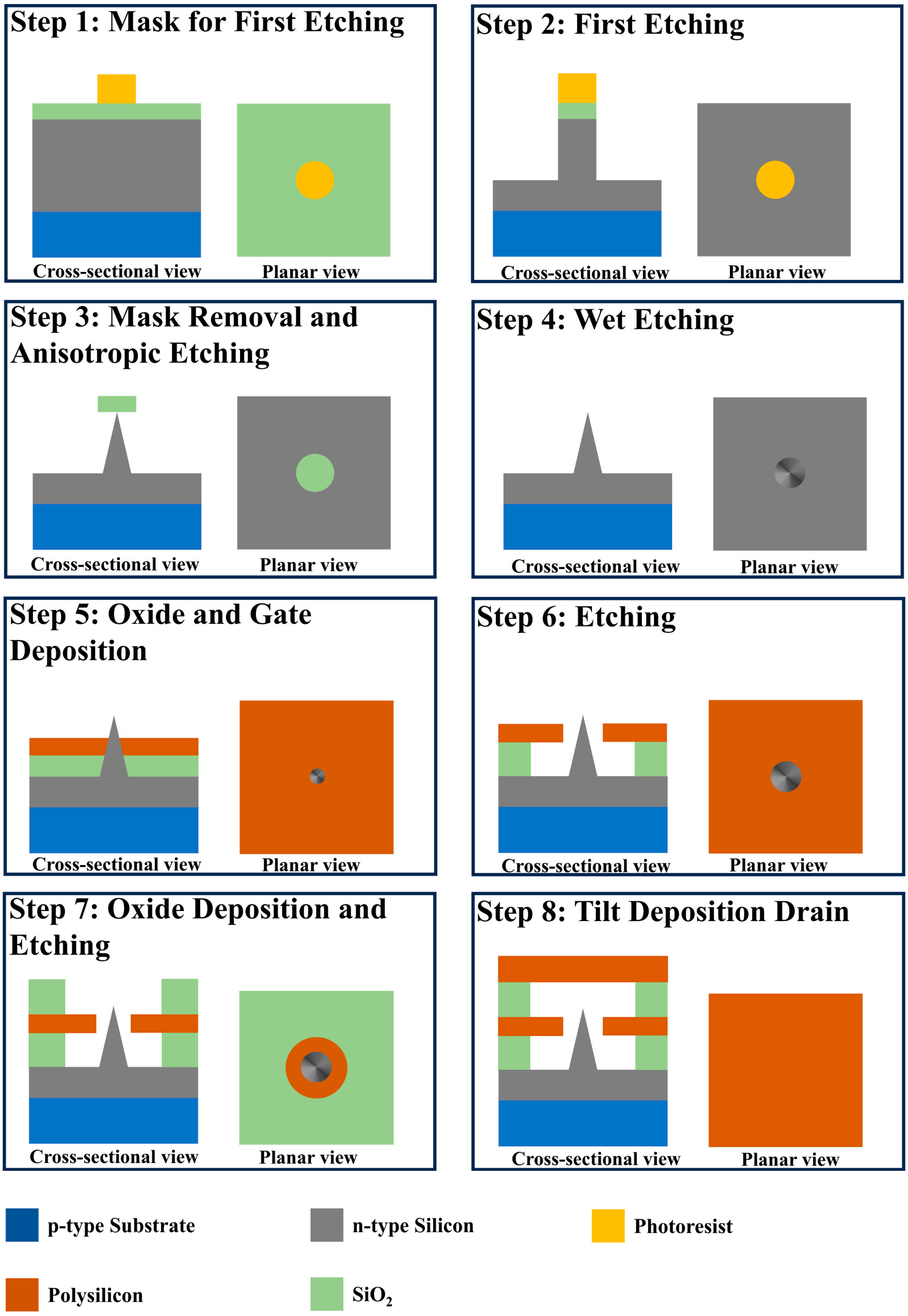

Figure S3. Schematics (not to scale) showing the Outlines of the fabrication process of the proposed VFET.

Reference

1. Heo, S.J.; Shin, J.H.; Jun, B.O.; Jang, J.E. Vacuum Tunneling Transistor with Nano Vacuum Chamber for Harsh Environments. *ACS Nano* **2023**, *17*, 19696-19708, doi:10.1021/acsnano.3c02916.
2. Shih, P.-C.; Perozek, J.; Akinwande, A.I.; Palacios, T. Anode-*Integrated GaN Field Emitter Arrays* for Compact Vacuum Transistors. *IEEE Electron Device Lett.* **2023**, *44*, 1895-1898, doi:10.1109/LED.2023.3317071.
